# Tissue Engineered Neural Constructs Composed of Neural Precursor Cells, Recombinant Spidroin and PRP for Neural Tissue Regeneration

**DOI:** 10.1038/s41598-019-39341-9

**Published:** 2019-02-28

**Authors:** V. P. Baklaushev, V. G. Bogush, V. A. Kalsin, N. N. Sovetnikov, E. M. Samoilova, V. A. Revkova, K. V. Sidoruk, M. A. Konoplyannikov, P. S. Timashev, S. L. Kotova, K. B. Yushkov, A. V. Averyanov, A. V. Troitskiy, J.-E. Ahlfors

**Affiliations:** 1 Federal Research and Clinical Center of Specialized Medical Care and Medical Technologies FMBA of Russia 28 Orekhovy Blvd., 115682 Moscow, Russia; 2 Scientific Center “Kurchatov Institute” - Research Institute for Genetics and Selection of Industrial Microorganisms”, 1-st Dorozhniy pr., 1, 117545 Moscow, Russia; 3 Federal Research Center “Crystallography and Photonics”, Institute of Photonic Technology of the Russian Academy of Sciences, 2 Pionerskaya St., Troitsk, 142190 Moscow, Russia; 4 Institute for Regenerative Medicine, I. M. Sechenov First Moscow State Medical University, 8 Trubetskaya St., 119991 Moscow, Russia; 5 N.N.Semenov Institute of Chemical Physics, 4 Kosygin St., 119991 Moscow, Russia; 6 National University of Science and Technology “MISIS”, 4 Leninsky Prospekt, 119049 Moscow, Russia; 7 New World Laboratories Inc., Laval, Quebec, Canada

## Abstract

We have designed a novel two-component matrix (SPRPix) for the encapsulation of directly reprogrammed human neural precursor cells (drNPC). The matrix is comprised of 1) a solid anisotropic complex scaffold prepared by electrospinning a mixture of recombinant analogues of the spider dragline silk proteins – spidroin 1 (rS1/9) and spidroin 2 (rS2/12) - and polycaprolactone (PCL) (rSS-PCL), and 2) a “liquid matrix” based on platelet-rich plasma (PRP). The combination of PRP and spidroin promoted drNPC proliferation with the formation of neural tissue organoids and dramatically activated neurogenesis. Differentiation of drNPCs generated large numbers of βIII-tubulin and MAP2 positive neurons as well as some GFAP-positive astrocytes, which likely had a neuronal supporting function. Interestingly the SPRPix microfibrils appeared to provide strong guidance cues as the differentiating neurons oriented their processes parallel to them. Implantation of the SPRPix matrix containing human drNPC into the brain and spinal cord of two healthy *Rhesus macaque* monkeys showed good biocompatibility: no astroglial and microglial reaction was present around the implanted construct. Importantly, the human drNPCs survived for the 3 month study period and differentiated into MAP2 positive neurons. Tissue engineered constructs based on SPRPix exhibits important attributes that warrant further examination in spinal cord injury treatment.

## Introduction

Spinal cord injury (SCI) is one of the most difficult-to-treat conditions with an incidence ranging from 250 to 906 cases per million per year worldwide^1^. In spite of a large arsenal of neuro-rehabilitation tools and procedures that include reconstructive spinal surgery and mechanical rehabilitation, kinesiotherapy as well as magnetic and electric stimulation, the degree of spinal cord function restoration in severe spinal cord trauma (American Spinal Injury Association, ASIA A or B level) is still catastrophically low^2,3^.

One of the most promising experimental approaches for spinal cord regeneration is transplantation of neural stem cells or neural precursor cells (NPCs)^4–7^. The development of safe techniques to generate autologous NPCs, first suggested by the use of iPS technology^8,9^, and later by direct reprogramming of somatic cells^10–13^, opened up new therapeutic opportunities in the regenerative field. In particular, the use of autologous NPCs prepared from the patients’ bone marrow with the help of a cocktail of non-integrating transcription factors and small molecules (without viral transduction, pluripotency factors or any other gene engineering procedures), demonstrates the greatest potential from a clinical viewpoint. drNPC (directly reprogrammed Neural Precursor Cells)^13–15^ are non-immunogenic, have a stable genome and pose a minimal risk of malignant transformation, especially when compared to iPS and embryonic stem cells; moreover, they exhibit the expected characteristics of neural stem cells such as self-renewal and multipotency.

A meta-analysis of more than 70 preclinical studies on allogeneic neural stem cells or NPC transplantation in spinal cord injury models has shown that combination of cell therapy with various scaffolds improves function restoration when compared to cell therapy alone^16^. Enhanced survival and differentiation of NPCs in the context of a preconditioned tissue engineered scaffold is one of the factors underlying the increased efficacy reported. Various types of materials have been used with the objective of boosting NPC efficacy: hydrogels^17,18^, including those based on modified fibrin^19^, collagen and other proteins of the extracellular matrix^20–22^, self-assembling peptides^23^ and biomimetic nanofibrous materials^24^.

Polysaccharides and proteins obtained from animals that are phylogenetically very different from humans have found surprising applications as scaffolds for tissue engineering. For instance, the spider dragline silk protein, spidroin (Major ampullate spidroin 1, MaSp1) has been shown to facilitate adhesion of NPCs through the activation of neural cell adhesion molecule (NCAM) expression and promotes neural differentiation due to the expression of multiple repeats of a neuron-specific GRGGL sequence recognized by neural progenitors^25^. Recombinant analogues of natural spidroin 1 and spidroin 2 (rS1/9 and rS2/12 proteins, respectively) are characterized by high biocompatibility, absence of immunogenicity, low biodegradability and ability to promote animal tissue regeneration^26,27^.

Another scaffold component of interest in regeneration is platelet-rich plasma (PRP). A number of studies have shown that PRP promotes neurogenesis and axonal growth^28^, as well as enhancement of proliferation and migration of Schwann cells^29^ due to the presence of different growth factors (PDGF-AB, TGF-β1, IGF-1, VEGF, NGF and GDNF) and platelet-released exosomes containing microRNA and other signaling molecules. These properties make PRP a prospective source of autologous growth factors providing the necessary biomimetic microenvironment for neuroregeneration^30^.

The aims of this study were to (1) develop a two-component matrix SPRPix, based on PRP and an anisotropic complex scaffold of recombinant spidroins and polycaprolactone (rSS-PCL) for the creation of 3D tissue engineered constructs with drNPC (2) to test the biocompatibility of the drNPC-SPRPix construct in the brain and spinal cord of the *Rhesus macaque* and 3) to examine the survival and behaviour of the implanted human drNPC cells.

## Results

### Immunophenotyping of drNPC

**Figure 1:**
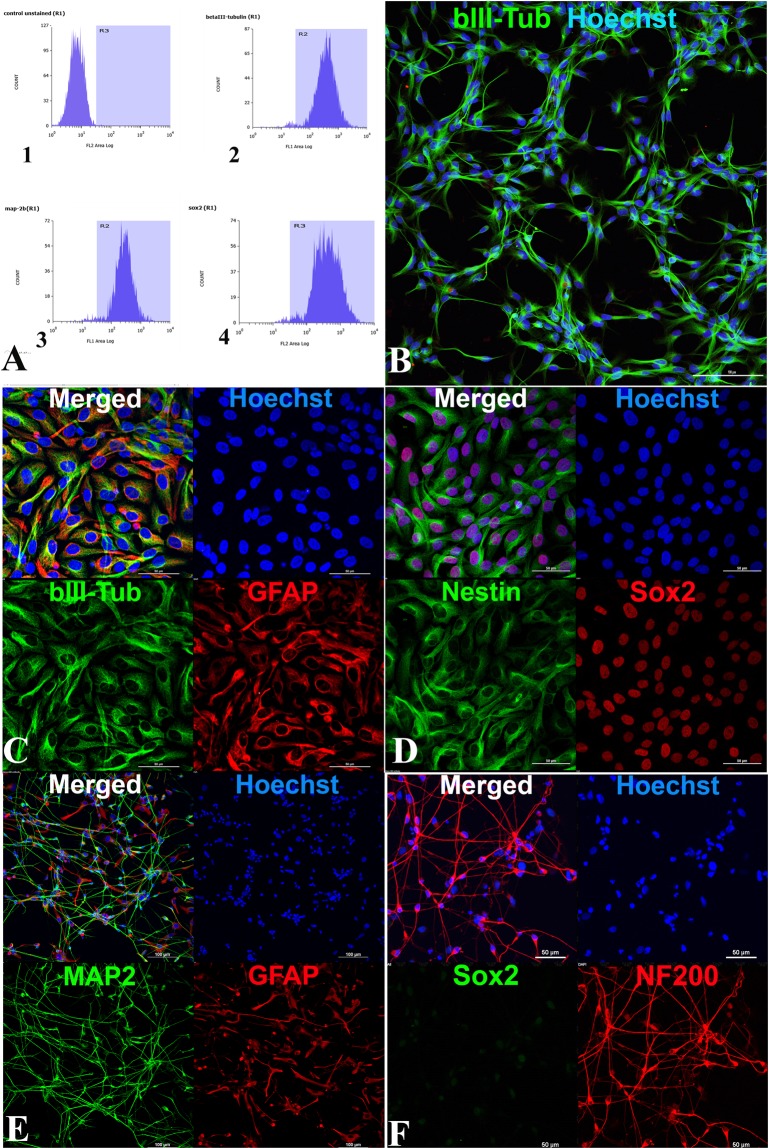
Immunophenotyping of drNPC. (**A**) Cytofluorometry of drNPC marker expression. 1. Negative control (isotypic immunoglobulins) 2. βIII-tubulin 3. MAP2 and 4. SOX2. (**B**–**F**) Immunocytochemical analysis for different markers (**B**) anti-βIII-tubulin. Bar = 100 µm (**C**,**D**) Undifferentiated drNPC coexpress βIII-tubulin (green) and GFAP (red) (**C**), as well as Nestin (green) and SOX2 (red) (**D**). (**E**,**F)** Differentiation of drNPC: MAP2-positive neurons (green) and GFAP-positive astrocytes (red) (**E**) NF200-positve (red) and SOX2 negative neurons (**F**). In panels (B–F), the blue channel corresponds to the Hoechst stained cell nuclei. In panels С–F Bar = 50 µm. Laser scanning confocal microscopy using Nikon A1 device.

We initiated our study by characterizing the drNPCs obtained from direct reprogramming of human bone marrow cells^13,15^. Using flow cytometry and immunocytochemical analysis, we demonstrated high level expression of the neural stem cell marker SOX2 and neural markers βIII-tubulin and MAP2 in the drNPC culture (Fig. 1A). drNPC cultured on laminin-coated adhesive plastic in complete medium exhibited a characteristic “honeycomb” pattern and co-expressed βIII-tubulin and GFAP (Fig. 1B,C), SOX2 and Nestin (Fig. 1D). Upon switching to differentiation conditions, MAP2- and NF200-positive neurons emerged (Fig. 1E,F), as well as GFAP-positive astrocytes (Fig. 1E). Differentiated cultures did not express SOX2 (Fig. 1F), indicating complete differentiation. Thus, the flow cytometry and immunocytochemical analysis confirmed that drNPC cells have a human neural stem like phenotype and were capable of differentiation into both neuronal and glial lineages.

### Characterization of drNPC cultures in a PRP-based “liquid matrix”

**Figure 2:**
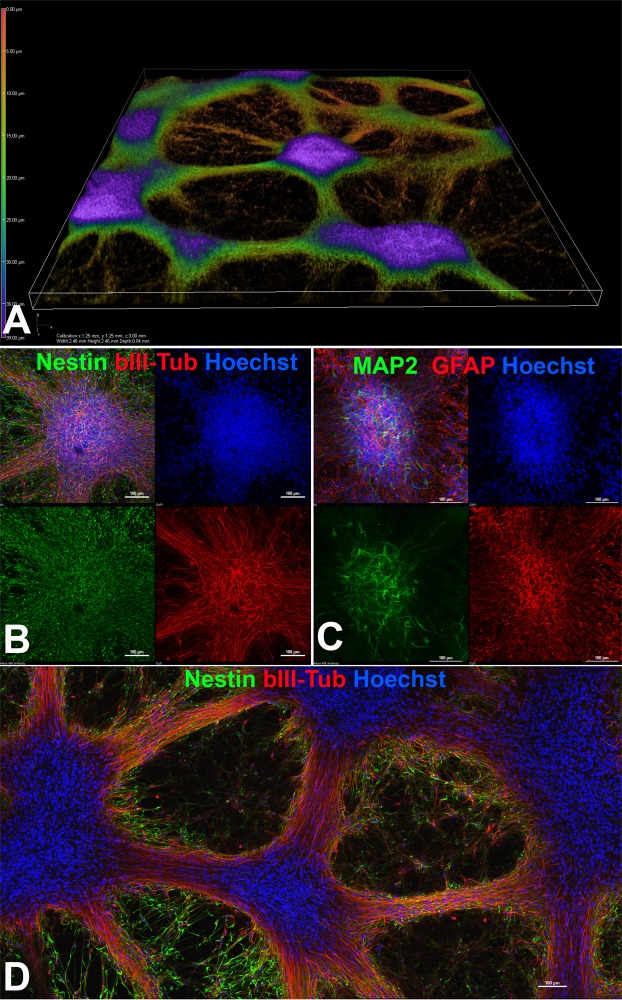
drNPC cultured in the PRP based liquid matrix. (**A**) 3D reconstruction of cultured drNPCs-01 in PRP based hydrogel using laser scanning confocal microscopy. (**B**) Spontaneous differentiation of neural organoids formed in PRP based hydrogel with the appearance of βIII-tubulin neuronal progenitors (red) amongst the more common Nestin positive (green) NPCs (**C**) Neural organoids stained with MAP2 (green) and GFAP (red) further confirming glial and neuronal differentiation. (**D**) Low magnification image showing the overall morphology of the differentiating cultures with large numbers of Nestin positive (green) NPCs and βIII-tubulin positive neurons (red). The optical plane cuts through the center of the neural organoids. (**B–D**) bar = 100 µm.

Having characterized the drNPC during active expansion and upon differentiation in adhesive plastic, we then characterized the behavior of these cells upon culture in the new PRP-based “liquid matrix”; after 2–3 weeks of culture we observed an almost complete replacement of the PRP hydrogel by a three-dimensional neural network that consisted of differentiating neural organoid-like structures, interconnected by processes of differentiating βIII-tubulin and MAP2-positive neuronal progenitors (Fig. 2A). Nestin- and GFAP-positive glial progenitors were distributed rather uniformly in the differentiating neural organoids, while βIII-tubulin and especially MAP2-positive young neurons had a more central localization (Fig. 2В,C). The long processes of βIII-tubulin positive neurons were surrounded by nestin-positive progenitors (Fig. 2D). In conclusion, the PRP-based liquid matrix preserves the biological attributes of the cells, supporting proliferation and spontaneous differentiation into neuronal and glial lineages.

### drNPC culturing on low-adhesive plastic coated with recombinant spidroin rS1/9

With the objective of testing the compatibility of drNPCs with spidroins, we then seeded drNPCs on a plastic dish coated with rS1/9 which contains the neural cell adhesion molecule (NCAM) specific amino acid sequence^25^. The cells rapidly adhered not only to the plastic but also to a glass coverslip in the center. Despite the use of complete medium, drNPCs plated on rS1/9 coated plastic exhibited intense spontaneous neuronal differentiation with the formation of βIII-tubulin positive neuroblasts and immature neurons (Suppl. Figs 1 and 2). This was accompanied by a high expression of BDNF as shown by immunocytochemical analysis (Suppl. Fig. 2). These data demonstrated that spidroin rS1/9 promoted not only drNPC adhesion to culture surfaces but also active neuronal differentiation, which we could partially replicate in laminin-coated plastic but only in the presence of differentiation medium.

### Construction and testing of various scaffolds

Having determined that spidroin rS1/9 had no negative impact on the biology of drNPCs, we then started working on constructing a spidroin-based scaffold. In parallel we also tested a commercially available collagen scaffold «Chondro-Gide»^®^ (Geistlich Pharma AG, Switzerland) widely used for cartilage tissue regeneration, as a model system to develop the technique for creation of a two-component matrix based on PRP and a solid scaffold.

We tested a complex scaffold prepared by electrospinning a mixture of recombinant spidroins and polycaprolactone (PCL, a biocompatible and biodegradable compound with a MW ~ 400 kDa) – we called this scaffold rSS-PCL. Spiders are known to use a mixture of spidroins 1 and 2 to boost the resilience of the dragline silk of a web, the first of which confers enhanced strength and the second one, due to the presence of a GPGXX (X = G,Y,Q) motif, confers enhanced elasticity^31^. In order to benefit from both properties, we utilized a mixture of rS1/9 and rS2/12 proteins. PCL was added to the mixture to improve its spinnability. We experimentally determined that a ratio of 6:1:1 (rS1/9:rS2/12:PCL) resulted in the optimal combination of scaffold stiffness and wettability which resulted in improved proliferation and neuronal differentiation of drNPC as shown in the corresponding results section.

### Physico-chemical characterization of the rSS-PCL scaffold

**Figure 3:**
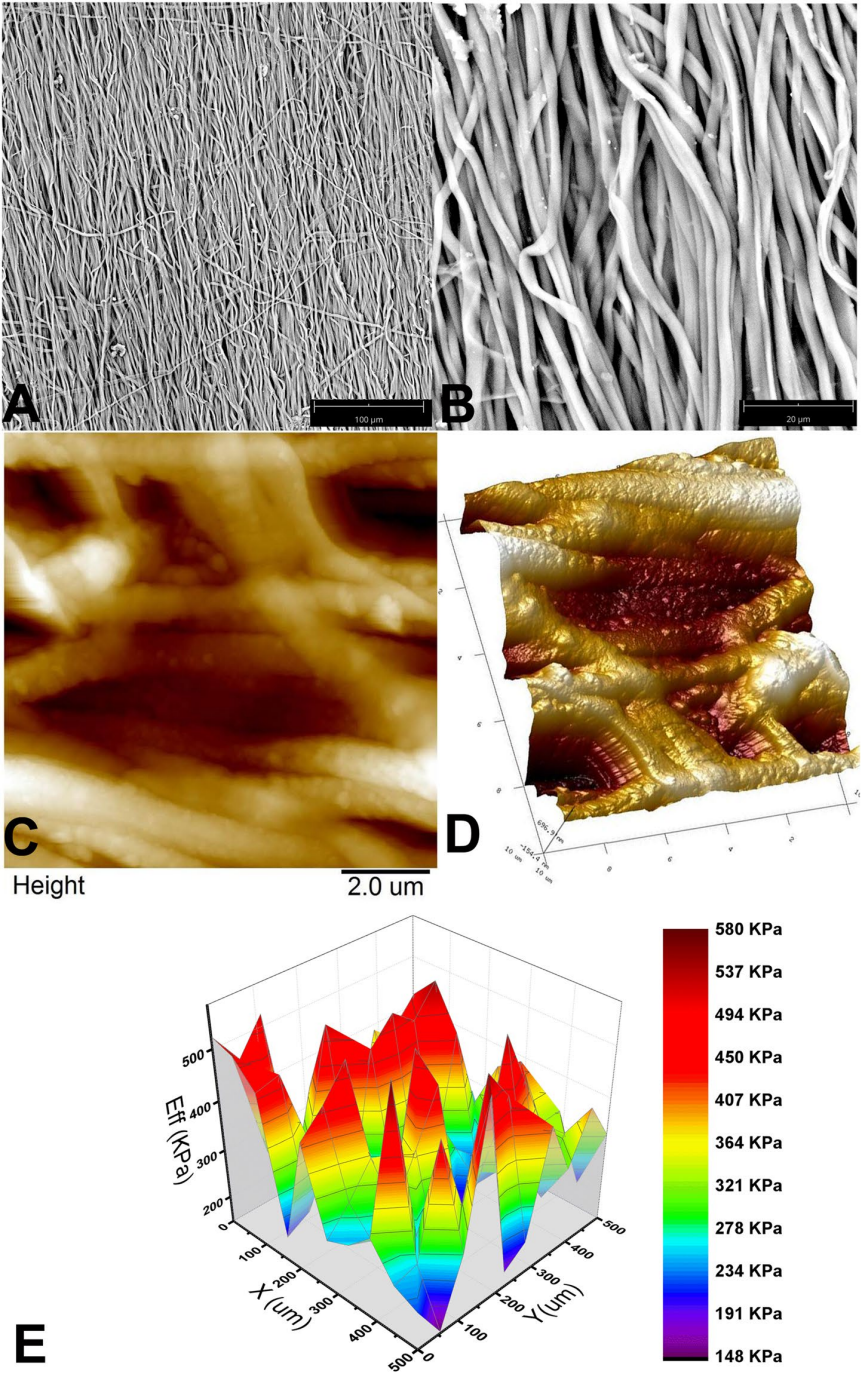
The spatial architecture and mechanical properties of the **rSS-PCL** scaffold: (**A**,**B**) SEM images, (**C**), 2D and (**D**), 3D – atomic force microscopy images, (**E**). typical Young’s modulus distribution obtained by nanoindentation.

Scanning electron microscopy (SEM) demonstrated that the rSS-PCL scaffold was characterized by an anisotropic structure with a predominantly unidirectional orientation of parallel microfibrils; the thickness of each microfibril was about 1 µm (Fig. 3A,B). More detailed imaging with Atomic Force Microscopy (AFM) (Figs 3C and 5D) revealed that the microfibrils were not smooth, and their surface had a considerable roughness with a nanodimensional grain structure. Such a combination of micro- and nanotopography of the surface of spider silk-proteins is considered ideal for culturing eukaryotic cells^32–35^.

The Young’s modulus of the rSS-PCL scaffold in the hydrated state, measured by nanoindentation, was found to be 287 ± 32 kPa (Fig. 3E) and did not change after the scaffold was pressed under a 2 ton weight, which indicates an extraordinary ability of the material to sustain its spatial architecture and mechanical properties. Finally, studies of the wetting properties of the rSS-PCL scaffold demonstrated good hydrophilicity, with an equilibrium contact angle of 63° ± 7 °C.

Taken together, the physico-chemical properties of the rSS-PCL scaffold (unidirectionally oriented structure, developed micro- and nanotopography, appropriate elasticity and integrity) support its use as a carrier for a growing neuronal network.

### drNPC culture in Chondro-gide® and SPRPix matrix

When drNPC were placed into Chondro-Gide^®^ in complete medium alone, good cell adhesion to collagen fibers was observed; however, the proliferative activity and neural differentiation of the cultured drNPC was very low (Suppl. Fig. 2А). Seeding the same number of drNPC onto Chondro-Gide^®^ in a mixture of PRP and complete medium, with the subsequent gel-clot formation on the scaffold surface, promoted a substantial increase in both proliferation and neural differentiation of drNPC in the gel phase of the two-component matrix (Suppl. Fig. 2B). Processes of differentiated neuronal progenitor cells were observed along the collagen fibers (which had a random spatial orientation).

**Figure 4:**
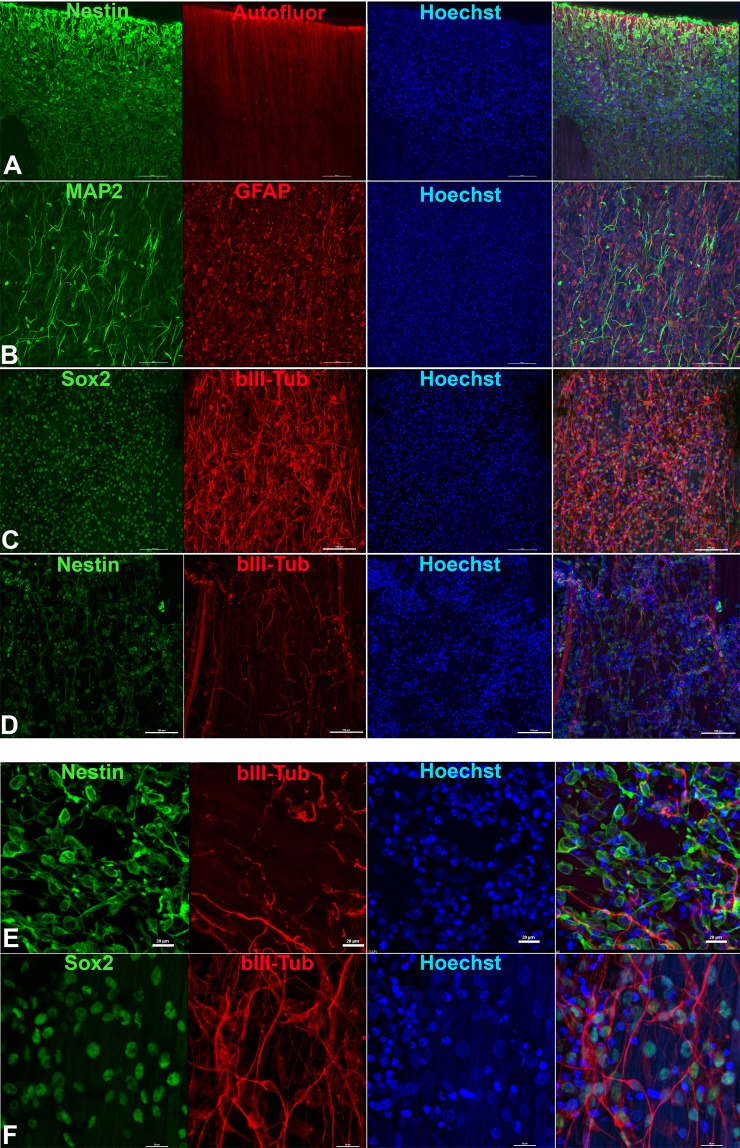
drNPC cultured in the rSS-PCL scaffold with or without the PRP-based liquid matrix (SPRPix matrix). (**A–C**) SPRPix (PRP + rSS-PCL scaffold); (**A**) Nestin (green), auto-fluorescence of the SPRPix matrix (red). (**B**) Spontaneous neuronal and glial differentiation revealed by MAP2 (green) and GFAP (red). (**C**) Intense proliferation of SOX2-positve (green) drNPCs, and βIII-tubulin-positive (red) neuronal progenitors. (**D**) The same concentration of drNPC cells seeded on rSS-PCL scaffold only. Nestin (green), βIII-tubulin (red). (**E**,**F**) high magnification examples of drNPCs cultured on rSS-PCL alone (**E**) or on SPRPix (**F**). Bar = 50 μm.

In contrast to what was observed in Chondro-Gide^®^, drNPC cultured on the two component SPRPix matrix (PRP hydrogel embedded into the rSS-PCL scaffold) demonstrated a high level of adhesion and neuronal differentiation: drNPC completely covered the entire SPRPix matrix surface and formed long βIII-tubulin- and MAP2- positive processes oriented along the spidroin microfibrils (Fig. 4A–C,E). In contrast, omission of the PRP hydrogel from the culture led to the formation of drNPC “islets” with short, scarcely oriented processes (Fig. 4D,F).

When compared with Chondro-gide^®^ plus PRP, the SPRPix matrix further increased both the proliferative activity of the seeded drNPC and the fraction of cells that underwent neuronal differentiation. Using the NIS Elements software (Nikon), we counted the total cell number (via the Hoechst channel) and the number of neuronal progenitors within the 640 × 640 × 25 µm (x × y × z) volume. The analysis of the ratio of Hoechst-positive cells (total number of cells) and βIII-tubulin positive cells (neuronal progenitors) showed that, in the case of the SPRPix matrix, the absolute numbers of cells/neuronal progenitors in the studied volume were 2600 ± 364/930 ± 148 after two weeks of culture, as compared to 1850 ± 296/174 ± 29 when cells were plated in rSS-PCL scaffold only (ie, without PRP). Thus, the total number of attached viable cells was 1.4 fold higher for the two-component SPRPix matrix than the single-component rSS-PCL scaffold (p < 0.05 by the Student’s t-test), while the fraction of neuronal progenitors was 3.8 times higher (9.4% vs 35.8% for the rSS-PCL scaffold and SPRPix matrix, respectively) (Suppl. Fig. 4). The absolute number of neuronal progenitors in the rSS-PCL scaffold and SPRPix matrix differed by more than 5 times (p < 0.01 by the Student’s t-test).

### Neurite alignment estimation in SPRPix matrix images by Fourier-transform statistical analysis

**Figure 5:**
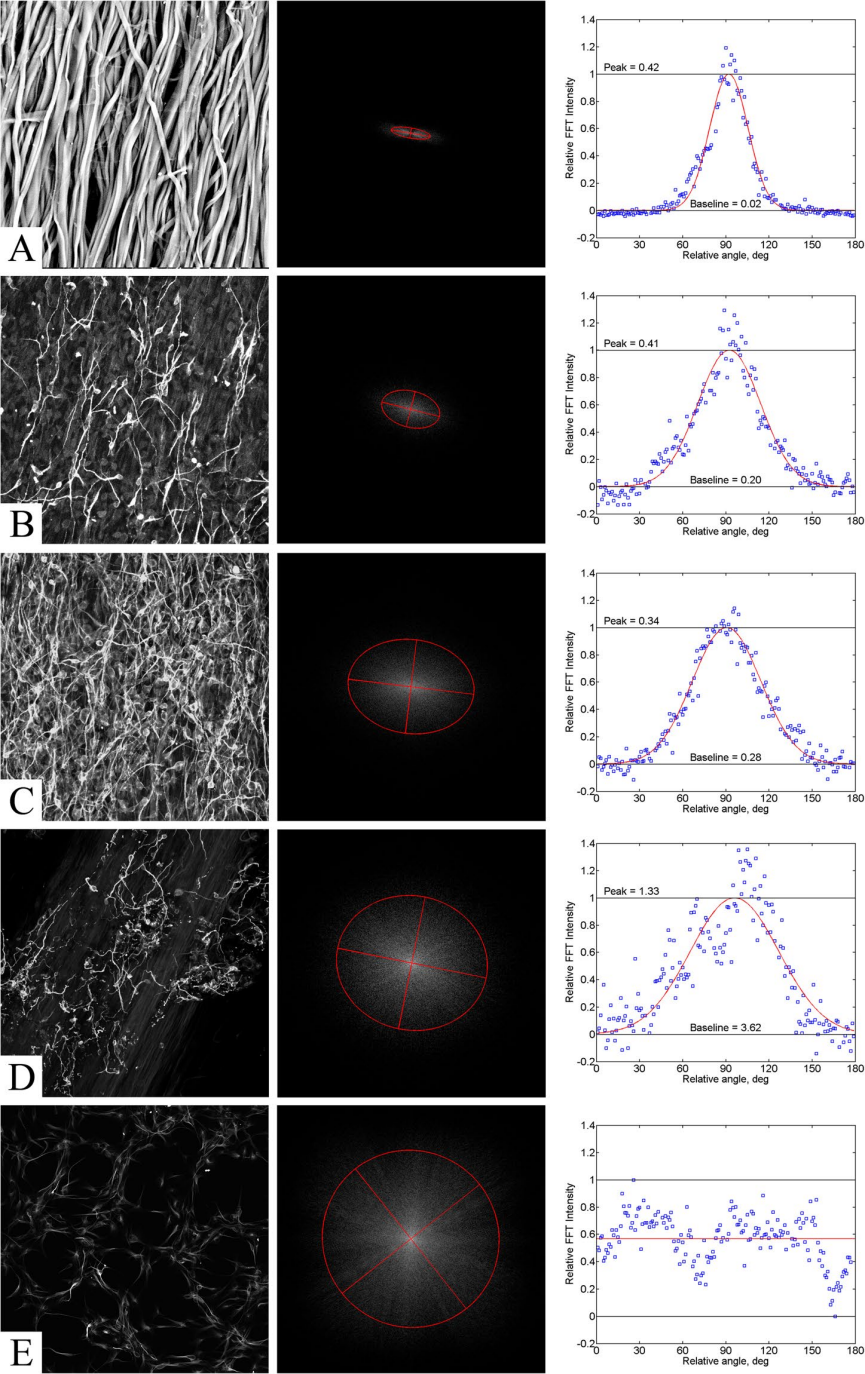
Analysis of structure alignment using image FFT and second order moments. (**A**) SEM of rSS-PCL scaffold; (**B**). MAP2 staining; drNPC in SPRPix matrix (rSS-PCL + PRP); (**C**). βIII-tubulin staining; drNPC in SPRPix matrix; (**D**). βIII-tubulin staining; drNPC in rSS-PCL scaffold; (**E**). βIII-tubulin staining; drNPC cultured on isotropic surface. **Left** – original grayscale images; **Middle** – FFT images with equivalent covariance ellipses; **Right** – angular distribution of FFT intensity with normal fit.

In order to study neurite orientation in the SPRPix matrix, we analysed βIII-tubulin-positive cells using FFT statistics (summarized in Fig. 5). The first column shows original grayscale images used for statistical analysis. The second column shows the resulting FFT images (Fig. 5A). The intensity of these images is shown in a logarithmic scale linearly fitted to the [0,1] interval. The red lines in the plot show equivalent covariance ellipses. The third column shows a normalized angular distribution of FFT image sector sums fitted with a normal distribution.

The results of directionality analysis are summarized in Supplemental Table 1. The SEM image of the rSS-PCL scaffold (Fig. 5A) shows high directionality with *ɛ* close to 1 and standard deviation *σ* below 30°. All images of neurite structures grown on anisotropic scaffolds demonstrate noticeable directionality with eccentricity form 0.46 to 0.79 and corresponding standard deviation between 40° and 50°. Higher standard deviation σ values were observed for the two-component SPRPix scaffold (Fig. 5B,C) compared to that of anisotropic rSS-PCL scaffold (Fig. 5D). The control image of drNPC without any scaffold shows the lowest eccentricity *ɛ* < 0.2. Taken together this data shows that the degree of neurite orientation is statistically higher on anisotropic scaffolds compared to no-scaffold control which shows almost isotropic growth (Fig. 5E).

### *In vivo* study of biocompatibility of SPRPix matrix in nonhuman primates

**Figure 6:**
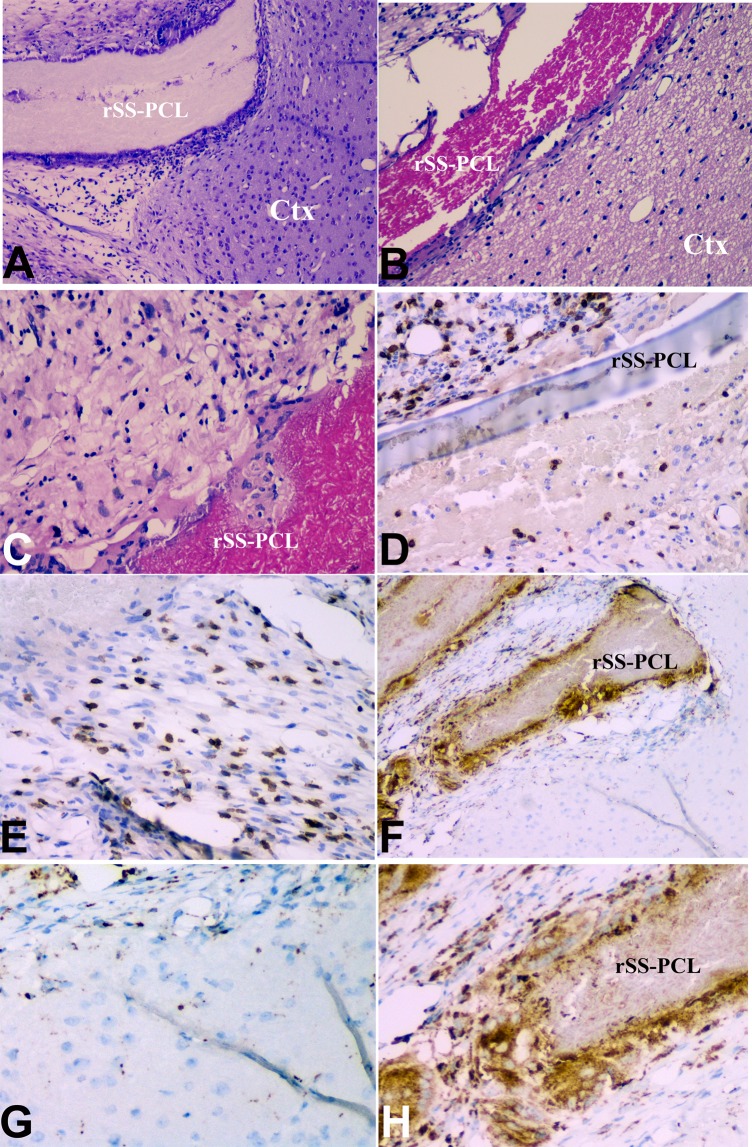
Histological and immunohistochemical (IHC) analysis of the endogenous immune response to the drNPC-SPRPix implanted into the brain cortex of a rhesus macaque. (**A**) Nissl stain; magnification ×100 (**B**,**С**). H&E stain of the interface between the SPRPix and the surrounding host tissue; magnifications ×100 (**B**) and ×200 (**С**). (**D**,**E**). Representative images of CD3 staining showing low level of T cell infiltration in the brain tissue adjacent to the drNPC-SPRPix implant. (**F**–**H**) Representative images of CD68 staining showing examples of microglial/macrophages in the brain adjacent to the drNPC-SPRPix implant (**G**) and among the human cell graft (**H**). (**E**,**H**) depict the areas with the highest immune cell infiltration noted. Bar size 100 µm. Notations: rSS-PCL-spidroin-polycaprolactone scaffold; Ctx – brain cortex.

**Figure 7:**
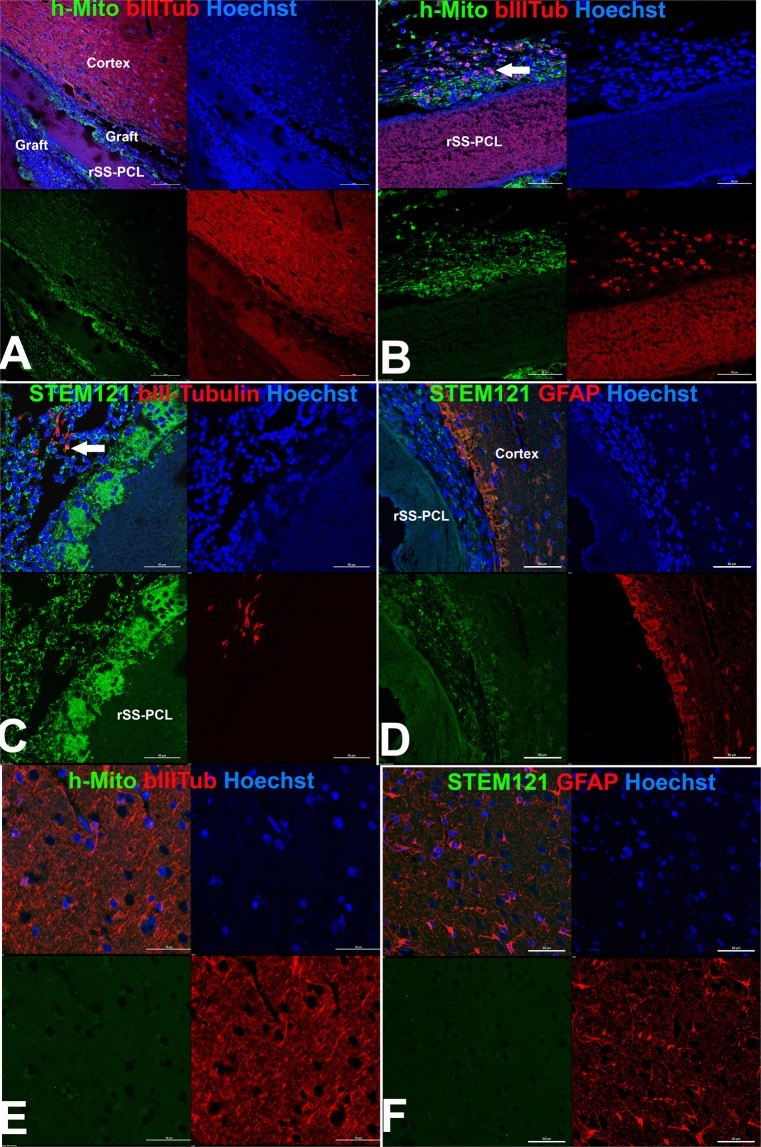
Immunofluorescence analysis of the brain of a rhesus macaque at the site of implantation of the SPRPix matrix with human drNPC. (**A**) Representative low magnification image of the site of drNPC-SPRPix implantantation showing staining by human specific anti-mitochondria antibodies (h-Mito) and some co-expression with βIII-tubulin. (**B**) Higher magnification of h-Mito and βIII-tubulin staining. Arrow shows co-localization of h-Mito and βIII-tubulin indicating neuronal differentiation of transplanted human drNPCs. (**C**) Human specific staining by STEM-121 co-localized with anti-βIII-tubulin (arrow). (**D**) Staining with STEM-121 and anti-GFAP revealed no co-localization. (**E**–**F**) Staining of normal brain tissue shows absence of h-Mito (**E**) and STEM-121 (**F**) staining of non-tranplanted NHP host tissue. Bar 100 µm. Laser scanning confocal microscopy.

As a final experiment, we implanted the SPRPix matrix seeded with human drNPC into the cerebral cortex and spinal cord of two NHPs (non human primates). The animals tolerated the surgeries well and no adverse events were detected during the in-life portion of the study, up to 12 weeks post implantation. Histopathology revealed no findings of microglial infiltration or gliomesodermal reaction in the host brain (Fig. 6A–C). In addition, there was insignificant astroglial response (Fig. 7) as reactive astrocytes were almost absent. Further immunohistochemical analysis with T-cell and microglial cell/macrophage markers revealed only a few infiltrating CD3-positive T-cells (Fig. 6D,E) and CD68 positive microglial cells (Fig. 6F–H) near the SPRPix matrix. The surrounding neural tissue demonstrated no signs of immune cell and microglial infiltration (Fig. 6G). Taken together, these data support biocompatibility of the drNPC-SPRPix combo in the NHP brain and spinal cord.

In order to reliably identify the implanted cells, two strategies were used. First, given that human female cells were implanted in male NHP recipients, we used antibodies against the macro-H2A.1 histone, which specifically labels the X-inactivated chromosome in the nuclei of female cells^36^. Second, two human specific antibodies – one against mitochondria (h-Mito), and another against a cytoplasmic antigen (STEM-121) were used to reliably identify human cells in the NHP host.

We found numerous human female cells in the implanted NHP brain (Fig. 7). h-Mito positive (Fig. 7A,B) and STEM121 positive drNPCs (Fig. 7C,D) were detected in and around the SPRPix. Staining with βIII-tubulin indicated that the transplanted human cells differentiated along the neuronal lineage (Fig. 7A–C), while glial differentiation along the astrocytic lineage was rare (Fig. 7D). We further confirmed that the antibodies used to identify human cells did not react with naïve NHP brain (Fig. 7E,F).

**Figure 8:**
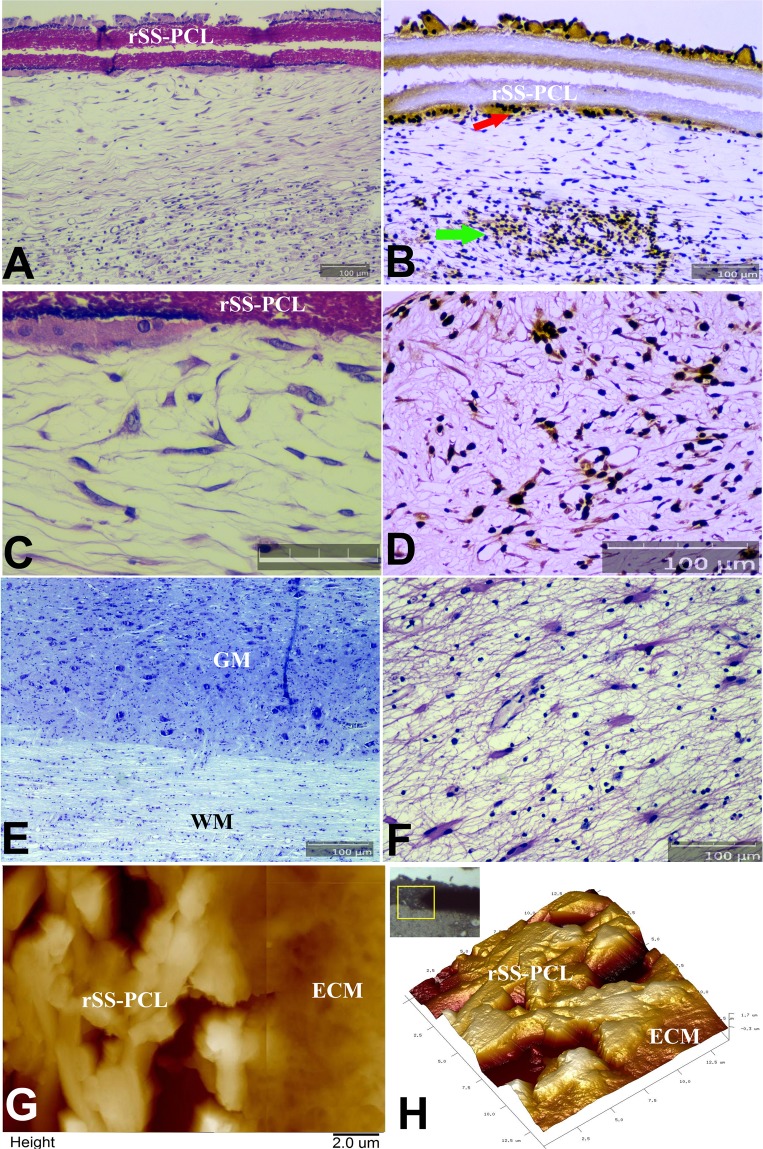
Histological and immunohistochemical (IHC) analysis of the SPRPix matrix with human drNPC implant with AFM imaging in the projection of the posterior column of the spinal cord of the rhesus macaque. (**A**) Sagittal section of the spinal cord stained with H&E. (**B**) Same spinal cord fragment, stained with antibodies to macro-H2A.1 visualized by immunoperoxidase staining. Transplanted cells highlighted by arrows. (**C**) Enlarged H&E image of (**A**) demonstrating the cells adjacent to the SPRPix matrix; Bar size = 50 µm. (**D**) Same image as in (**C**), stained with antibodies to MAP2. (**E**,**F**) Sagittal sections of the spinal cord directly below the SPRPix matrix, Nissl (**G**) and H&E (**H**), stains. (**G**,**H**) 2D AFM image (overlay of two images from adjacent regions) and a 3D AFM image of the border between the SPRPix and the ECM of the surrounding tissue, showing full integration of the scaffold with the ECM. In panels (**A**,**B**,**D**–**F**) bar size = 100 µm. Notations: rSS-PCL-spidroin-polycaprolactone scaffold; GM – grey matter; WM – white matter; ECM – extracellular matrix.

**Figure 9:**
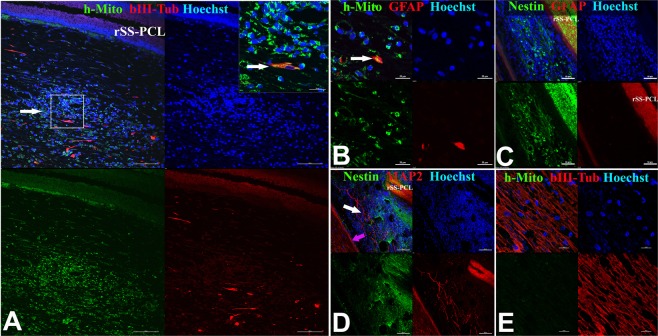
Immunofluorescence analysis of the spinal cord of a rhesus macaque at the site of implantation of the SPRPix matrix with human drNPC. (**A**) Representative low magnification image of the site of drNPC-SPRPix implantantation showing expression of human specific mitochondrial antigen (h-Mito) and βIII-tubulin (arrow) On the right: high magnification of the boxed area demonstrating co-localization of h-Mito and βIII-tubulin. (**B**) Rare example of colocalization of h-Mito with GFAP (arrow) at the site of transplantation. (**C**) Staining with human specific anti-Nestin and GFAP revealed numerous nestin-positive cells and the lack of astroglial reaction/differentiation. (**D**) Representative image of human specific anti-Nestin and MAP2. The white arrow points at the long MAP2-positive neurites derived from the human cells. The purple arrow shows the border of the spinal cord. (**E**) Control staining of naive spinal cord tissue demonstrated lack of h-Mito signal. Cell nuclei in all the images are stained with Hoechst (blue). Notations: rSS-PCL-spidroin-polycaprolactone scaffold; SC – spinal cord.

Very similar observations were recorded in the spinal cord by both histopathology (Fig. 8) and immunostaining (Fig. 9). In the implanted spinal cord, Nissl staining around SPRPix demonstrated lack of infiltration by host immune cells and histiocytes (Fig. 8A,C). No pathological changes were found neither in the white nor the gray matter directly adjacent to the implanted SPRPix matrix (Fig. 8A,C,E,F). In order to characterize the structure of the extracellular matrix surrounding the SPRPix, we performed an AFM study of the dried de-paraffinized sagittal spinal cord sections (Fig. 8G–H). This study showed that the SPRPix matrix was fully integrated within the surrounding tissue, and a loose, rather isotropic fine-fibered extracellular matrix (similar to that of normal neural tissue), was observed in the direct proximity of the SPRPix. This extracellular matrix did not contain any tightly packed oriented collagen fibrils characteristic of a fibrotic matrix or scar. Nissl staining also revealed some cells that morphologically resembled interneurons around SPRPix (Fig. 8C); further staining confirmed that these cells were MAP2 positive (Fig. 8D) and some of them were double positive for h-Mito and βIII-tubulin (Fig. 9A, Suppl. Fig. 6) which suggests that at least some of transplanted human cells migrated into the spinal cord tissue and differentiated into neurons.

Finally, additional immunofluorescence analysis revealed absence of GFAP-positive reactive astrocytes around SPRPix (Fig. 9B,C), where the presence of numerous human nestin-positive cells was noted (Fig. 9C,D). We also detected βIII-tubulin positive human neuronal progenitors (Fig. 9A) as well as MAP2-positive human neurons between the SPRPix matrix and the spinal cord, and these neurons extended long processes in the spinal cord (Fig. 9D).

## Discussion

Previous studies have demonstrated that transplantation of small (up to 500 µm) tissue engineered constructs outperform the transplantation of suspended cells alone based on a number of cell survival indices^16,18,22,24^. Various factors are known to underlie this observation: transplantation of tissue engineered constructs eliminates cell detachment stress due to the use of proteolytic enzymes and preserves cell-cell contacts that are important for cell survival and function. Furthermore, transplantation of cell constructs allows pre-differentiation of cells in culture, ensuring that the right cell phenotype is administered, presumably enhancing transplantation kinetics and functional outcomes.

Our work focused on developing a complex matrix with two highly relevant materials for culturing human NPCs and for implantation into the CNS: PRP and spidroins. Due to its growth and survival-rich factor content, PRP has been regarded as a potential substrate for the formation of 3D neural tissue engineered constructs^28,29^. PRP is also considered a biomimetic scaffold as it creates a natural microenvironment for NPCs, in which the cells, embedded in the hydrogel, may interact as they would in normal ontogenesis. Indeed, PRP-based biomimetic microenvironment is used to fill neural conduits during reconstructive surgery of peripheral nerve injuries^30^. Silkworm-produced silk fibroin and proteins of the dragline silk of orb-weaver spiders’ web – spidroins – are characterized by unique biomechanical and adhesive properties. Many researchers consider them as a prospective material for creation of recombinant tissue engineered scaffolds, including those for neural tissue^32–35^. Silk proteins have been mostly investigated for the development of scaffolds for regeneration of peripheral nerves^33,37^ and much less explored in the context of CNS regeneration. Interestingly, recombinant spidroin coating of cell culture surfaces has been shown to promote adhesion and neuronal differentiation^25^. In our study, the two materials were combined for the creation of a two-component scaffold based on PRP and an anisotropically structured spidroin scaffold.

We first showed that the “liquid matrix” consisting of 25% PRP hydrogel dramatically increased proliferation and neuronal differentiation of drNPCs both when culturing on a non-adhesive plastic and within the structure of the two-component SPRPix matrix, demonstrating its critical value as part of an engineered construct aimed at supporting regeneration of neural tissue. The observed stimulation of drNPC proliferation and neuronal differentiation in the SPRPix matrix is also likely due to the anisotropic rSS-PCL scaffold properties, including the presence of a large number of repeats (18 repeats) of the GRGGL sequence in rS1/9 that is a signal sequence for the growth of neural cells and serves as a ligand for binding with the NCAM receptors on the surface of neural cells^25^. The other favourable factor is the large positive net charge of the rS1/9 and rS2/12 molecules (+28 and +36 at рН 7.0, respectively) which facilitates adhesion of neural cells to the scaffold via the NCAM receptors. The high stiffness of the prepared scaffolds is also of great importance, since it is known to play a notable role in the proper differentiation of neural cells and axon growth, and the rather high hydrophilicity (65%), in spite of the presence of a more hydrophobic PCL, is significant as well.

In addition, the nanotopographical relief of the microfibril surface formed in the process of electrospinning is of particular importance for enhancement of biocompatibility of the 3D anisotropic spidroin scaffold^32,33^ as it facilitates efficient adhesion of neural progenitors and their processes on the scaffold surface, while the oriented architecture of microfibrils promotes oriented axon growth. The calculated alignment of the extended neurites, based on the Fourier-transform analysis of the microscopic images, shows that the axon growth is more oriented on the SPRPix matrix as compared to the components of PRP or rSS-PCL scaffold alone. This is probably due to the enhanced adhesion of neurites to anisotropic rSS-PCL provided by the PRP-based hydrogel.

When designing scaffolds for use in CNS tissue engineering a highly desirable feature is biocompatibility with nervous tissue. Spider silk spidroins are biocompatible and biodegradable and typically induce only a mild inflammatory response that decreases within a few weeks post-transplantation. It has been previously shown that, due to their biodegradability, recombinant spidroins can be remodelled in the body allowing vascularization, innervation and tissue ingrowth with the eventual complete replacement by the native bone and connective tissue^27^. Biocompatibility of recombinant spidroin-1 (known as rS1/9) has been shown in rodent experiments^25,27^. However, no studies have shown the biocompatibility of a scaffold containing recombinant spidroin in primates. Since we had combined both spidroin-1 and -2 (rS1/9 + rS2/12) into a single scaffold and further modified it with PCL (rSS-PCL), added a PRP hydrogel (that combined make up the SPRPix matrix), and seeded with human drNPCs, it was important to determine the biocompatibility of the resulting neural scaffold *in vivo*. Our study in two NHPs provided initial indications of biocompatibility and safety: the SPRPix matrix did not cause lymphocytic and microglial infiltration nor resulted in active astrogliosis and gliomesodermal reactions. Furthermore, the SPRPix matrix supported the survival of the transplanted human drNPCs within the engineered construct for at least 12 weeks post-transplantation. A subpopulation of the transplanted cells retained the expression of nestin and SOX2, while a significant portion of the cells became MAP2-positive neurons.

Our *in vivo* study did not employ the use of immunosuppressive drugs yet the human cells still survived in the NHP CNS. The absence of a notable reaction from the recipient immune system may be explained by low level of HLA-DR expression, weak antigen-presenting function, and/or the creation of a local immunosuppressive microenvironment. Indeed, iPS-derived neural stem/progenitor cells appear to exhibit these characteristics^38^ and the drNCP used in our study may behave in a similar way, with the additional advantage of being further protected from an initial host immune reaction due to their integration in a complex matrix. Future studies will investigate the mechanisms by which the SPRPix matrix promotes engraftment of drNPCs into the host CNS tissue without scar formation and minimal immune reactivity.

When addressing specifically the regeneration of the injured spinal cord, it is important to ensure not only the most favourable conditions for cell survival, but also for the promotion of oriented axonal growth. The SPRPix matrix appears to support both of these aspects *in vivo* upon implantation into the normal host spinal cord. This was achieved with minimal host immune response and no scar formation, demonstrating that the developed SPRPix matrix might be superior to other types of tissue engineered constructs like polysaccharide based hydrogel scaffolds^39^ and other polymeric scaffolds^40^. Further studies will investigate the efficacy of drNPC- seeded SPRPix in spinal cord injury models.

## Conclusion

The two-component SPRPix matrix described in this study, consisting of a “liquid” component based on a PRP-derived hydrogel within a rSS-PCL scaffold, dramatically stimulated proliferation and neuronal differentiation of drNPCs due to the complex action of the PRP components and creation of a 3D biomimetic environment with rSS-PCL that critically supported drNPC growth and differentiation. The rSS-PCL scaffold not only provided adhesion of drNPCs to the surface, but also functioned as a guiding scaffold, specifying a vector for axon growth resulting in aligned parallel neurite outgrowth. This study demonstrated the biocompatibility and safety of the two component SPRPix matrix in the NHP brain and spinal cord. Finally, our study provided preliminary evidence of integration and differentiation of the implanted NPCs into the host brain and spinal cord. The tissue engineered construct resulting from combining drNPC with the two-component SPRPix matrix exhibits important attributes that warrant further examination in spinal cord injury treatment.

## Materials and Methods

### Preparation of human neural precursor cells

Primary-reprogrammed human neural precursor cells (hereafter – directly reprogrammed neural precursor cells, drNPC) were provided by New World Laboratories Inc^13^. Cryopreserved cells were thawed from liquid nitrogen and seeded onto laminin-coated plates in NeuroCult-XF Basal Medium (StemCell Technologies) with 1% Pen-Strep (Gibco), B-27 Supplement (50×) (Gibco), 20 ng/mL of bFGF and 20 ng/mL of EGF (complete medium). The cells were incubated at 37 °С in 5% СО_2_ and 5% О_2_; medium replacement was performed every 2 days. As needed, cells were passaged at a ratio of 1:3 when 80% confluence was reached.

For differentiation studies, a standard differentiation protocol was used: drNPC monolayers were switched to Neurobasal media (Gibco, USA) supplemented by Glutamax (1×, Gibco, USA), B27 (1×, Gibco, USA), CultureOne (1×, Gibco, USA), BDNF (20 ng/ml), GDNF (20 ng/ml) and Ascorbic Acid (200 µM) for 14 days.

### Immunocytochemical analysis

The expression of neural stem cell, neuronal and glial markers was performed by immunocytochemical analysis. The cells were fixed by adding 4% buffered formaldehyde solution containing 0.1% saponin. Primary antibodies to Nestin (R&D, 2 μg/ml), SOX2 (BD Biosciences, 5 μg/ml), βIII-tubulin (R&D, 2 μg/ml), MAP2 (Sigma-Aldrich, 5 μg/ml), GFAP (DAKO, 5 μg/ml), and NF-200 (Sigma-Aldrich, 5 μg/ml) were used together with Alexa Fluor 488 goat anti-mouse IgG (H + L) and Alexa Fluor 633 goat anti-rabbit IgG (H + L) secondary antibodies (all 1:400; Invitrogen, USA). The cell nuclei were labeled with Hoechst 33342 stain (Thermo Fisher Scientific). Immunofluorescence was analyzed using a Nikon A1 scanning laser confocal microscope (Nikon Co., Japan). All the staining studies were conducted in series, with 5 repeats in each series.

### Spidroin coating of low-adhesive surfaces

drNPCs-01 were cultured on low-adhesive surfaces coated with spidroin (see biosynthesis details below) using Petri dishes with a cover glass bottom in the center (Confocal Petri Dishes, SPL Life sciences). For the cover glass coating with spidroin, 1% solution of the recombinant rS1/9 in 96% formic acid was poured into a sterile dish and incubated for 60 minutes, followed by the solution removal, washing of the dish with 96% ethyl alcohol and drying under UV-radiation in a laminar flow hood.

### Biosynthesis of the anisotropically structured complex spidroin matrix

The yeast biomass growth of *S*. *cerevisiae* SCR-702T-1F9 and SCR-702T-2EI2, and the extraction and purification of the recombinant spidroins rS1/9 and rS2/12, were conducted as previously described^41^. Briefly, the yeast cells were grown in a 3 L fermenter in YPD medium (2% of fermentative peptone (GOST 13805-76) and 1% of the yeast extract (Hefe-extract, Serva) in the presence of 2% glucose at 30 °С for 4 days.

To obtain the target proteins, 0.5 kg of the wet biomass was suspended in a lysis buffer (0.05 М sodium phosphate, рН 7.4; 0.001М EDTA; 5% glycerol) and the cells were lysed with glass beads in a MS_3 flow mill for 1.5 hrs; the prepared suspension was centrifuged, and the target protein was extracted from the precipitate by a 10% solution of lithium chloride in 90% formic acid with continuous stirring for 18 hrs followed by centrifugation. The supernatant was dialyzed against 10 mM sodium acetate solution at рН 4.0 and clarified by centrifugation. The final purification was conducted by ion-exchange chromatography using a HiPrep 16/10 SP FF (GE Healthcare) column on an ÄCTApurifier^TM^ (GE Healthcare) chromatograph with рН exchange (рН 4.0–рН 7.0 – pH 4.0). The protein was eluted with a NaCl concentration gradient, dialyzed against deionized water, frozen at −70С° and freeze-dried.

#### Preparing the anisotropically structured complex spidroin scaffold

rSS-PCL by electrospinning, a NANON – 01A apparatus equipped with a standard spinneret and a drum collector with a 100 mm width was used with a solution containing the recombinant spidroins rS1/9 (6%) and rS2/12 (1%), PCL (1%, «Biopolymer», Ukraine) in the ratio of 6:1:1, in 1,1,1,3,3,3–hexafluoro-2-propanol (HFIP; Fluka Chemie GmBH, Germany). The solution was fed at a rate of 0.4 mL/hr. The process was conducted at a voltage of 9.5 kV, with a collector rotation rate of 1000 rpm at a 10 cm distance from the capillary to the collector. When the process was finalized, the prepared material was incubated in 96% ethanol for 15 min, and then dried for 18 hrs at room temperature in a laminar flow hood.

### Preparation of PRP

All research involving human participants was performed in accordance with relevant guidelines/regulations. PRP was prepared from blood of three healthy male donors, after each donor had signed an informed consent for using their plasma for research purposes. The experimental protocol with the participation of blood donors was approved by the Local Ethics Committee of the Federal Research and Clinical Center of Specialized Medical Care and Medical Technologies of FMBA of Russia (Protocol No.10a of September 12, 2016). For preparation of PRP, we harvested 42 mL of donor blood into a syringe pre-filled with ACDA (acid citrate dextrose buffer Type A) (Hemocon) at a ratio of 1.4 mL per 10 mL. The harvested blood was centrifuged in an Eppendorf 5810 R centrifuge in 50 mL tubes (Corning) at 280 g and 20 °С for 15 min with acceleration of 3/3. Using a serological pipette, the upper 10–15 mL of plasma was collected above the erythrocyte level and the buffy coat, depending on the donor’s hematocrit, into a 50 mL tube. The sample was repeatedly centrifuged at 280 g and 20 °С, for 15 min; the upper two-thirds of the plasma was removed (Platelet poor plasma), the bottom one-third was re-suspended in the remaining volume, and the number of thrombocytes was estimated with a hemocytometer. We then selected PRP samples with the platelet count no less than 5 × 10^5^/µl.

### Cultivation of drNPCs in PRP-based “liquid matrix”

Monolayer drNPCs were removed from the surface of culture flasks by incubation with 2 mL of Trypsin-EDTA 0.25% (Gibco) after washing with Dulbecco’s phosphate-buffered saline (D-PBS) (Gibco). After the incubation, the trypsin-EDTA was inactivated with conditioned medium, and the suspended cells were transferred into a 50 mL tube, a cell count was performed using a TC20 automated cell counter (Bio-Rad), and the sample was centrifuged at 400 × g and 20 °С for 5 min. The supernatant was aspirated and the cell pellet re-suspended in 750 µL of the NeuroCult-XF Basal Medium (StemCell Technologies). 250 µL of the freshly prepared PRP was added to the cell suspension in NeuroCult-XF Basal Medium, resulting in a cell concentration of 4 million/mL that was then carefully re-suspended. To neutralize the sodium citrate (anti-clotting agent) in the Hemocon buffer, 2 µL of 10% CaCl_2_ per 100 µL of the medium was added and the solution immediately transferred into Petri dishes with a glass cover bottom (SPL Life sciences). The gel-clot formation occurred in the Petri dishes during 20 min at 5% СО_2_, 37 °С. Then, 1.5 mL of Complete medium was added to each Petri dish. The dishes were incubated at 5% СО_2_, 37 °С for 2 weeks, and the medium was replaced every 2 days.

### Preparation of two-component matrices from PRP and anisotropically structured complex scaffold rSS-PCL (SPRPix)

To create the two-component tissue engineered construct, the required size was cut with a scalpel from the sterile complex scaffold, prepared as described above, and placed in the center of a glass cover bottom Petri dish. 50 µL of the drNPC cell suspension (with a concentration of 4 million/mL), mixed with 25% PRP in the NeuroCult-XF Basal Medium containing CaCl_2_, prepared as described above, was placed in layers onto the scaffold and incubated in 5% CO_2_ at 37 °С until gel-clot formation. Then, 2.5 mL of Complete medium was added. The dishes were incubated at 37 °С in 5% СО_2_; medium was replaced every day.

Five independent batches of SPRPix were made; for each SPRPix batch, a total of five replicas were prepared. As a control we also used a commercially available collagen scaffold «Chondro-Gide»^®^ (Geistlich Pharma AG, Switzerland) which was seeded by the same number of drNPCs with or without PRP (50 µL of the drNPC cell suspension with a concentration of 4 million/mL), following by cultivating during 2 weeks before fixation and immunostaining.

### SEM, AFM and mechanical studies

#### AFM images of the anisotropic complex scaffold

Were acquired on air in the PeakForce Tapping® Mode, using a MultiMode 8 atomic force microscope with a Nanoscope V controller and E scanner (Bruker). AFM imaging was conducted with RTESPA-300 probes (Bruker) with a nominal spring constant of 40 N/m, nominal frequency of 300 kHz and a nominal tip radius of 8 nm. 10 × 10 µm images were obtained at a scan rate of 1 Hz and a 512 × 512 pixels resolution. The raw AFM images were processed using the NanoScope Analysis v.1.10 software (Bruker).

#### Mechanical studies

Of anisotropic scaffold microfibrils were performed using a PIUMA Nanoindenter (Optics11, Amsterdam, Netherlands), which included a controller, an optical fiber and a spherical probe for the force-displacement curves’ acquisition. The probe of a 26.5 µm radius was attached to a flexible cantilever with the spring constant of 0.44 N/m in contact with the optical fiber to measure its displacement.

Anisotropic scaffolds (4 samples in total) were studied in water over the area of 500 × 500 µm, with a 50 µm increment by each axis. To obtain the force-displacement curves, the probe was immersed for 5 µm into a scaffold sample at each point of measurement. The Young’s modulus for each point was computed according to the Hertzian contact mechanics model for a spherical body indenting a flat surface, using the built-in PIUMA software.

### Contact angle studies

In order to assess the wetting properties of the anisotropic scaffold, we conducted contact angle measurements. We applied the sessile drop technique at 25 °С and a relative humidity of 30% with an experimental optical unit described in^42^. A 50 µL drop of distilled water, stained with Bengal Rose (Acros Organics, India), was placed onto a studied sample’s surface. The contact angle measurements were performed in the continuous video regime for 300 seconds, with a total of 9 measurements.

### Fourier-transform image analysis

For a quantitative estimation of neurites’ alignment in the anisotropic rSS-PCL scaffolds we used a numerical analysis of the digital images obtained with SEM (for the scaffold alone) and with scanning laser confocal microscopy (for the tissue engineered constructs) using two-dimensional fast Fourier transform (FFT). The images were 8-bit grayscale TIFF files. All files were cropped to the same size of 825 × 825 pixels. A custom script in MATLAB (MathWorks Inc., USA) was written for the image analysis, allowing FFT of grayscale images to be generated by the *fft2* function producing a complex-valued matrix of the same size as the original image. Modulus of FFT was treated as a probability distribution function in the frequency domain, and its asymmetry characterizes alignment of structures in the image^43^. For a higher accuracy of statistical image analysis we reduced the noise of FFT applying a cut-off to the FFT matrix: all values below a preset limit were zeroed. The limit has been chosen based on the noise level estimated from the maximum values of 64 × 64 pixel subarrays in the corners of the whole FFT matrix. Second-order statistical moments were used to characterize the asymmetry of FFT distributions^44^. Three independent second-order central moments determine an equivalent ellipse in the frequency domain. The shortest axis of the ellipse indicates the prevailing direction of the structure alignment in the original image (mean value *μ* of angular distribution). Eccentricity of the ellipse *ɛ* is a measure of alignment dispersion. For a randomly oriented structure eccentricity tends to 0; for a totally aligned structure eccentricity approaches 1. The main numerical measure of alignment is the angular standard deviation *σ*. In this experiment, *σ* was calculated using a weighted sample variance of normal distribution fitting the dataset. These calculations were performed for each series of experiments.

### *In vivo* study of biocompatibility on healthy primates

The experimental protocol for the study of biocompatibility of the neural tissue engineered SPRPix constructs containing human drNPC on healthy nonhuman primates was approved by the Local Bioethical Committee of the Research Institute of Medical Primatology of Russian Academy of Sciences (Protocol of July 13, 2016). The experiment was conducted on two healthy male rhesus macaques (*Macaca mulatta*) born in 2012 (Table 1) homed at the farm of the Research Institute of Medical Primatology of Russian Academy of Sciences in Sochi. One month prior to the study, the animals were relocated from the shared cage into individual cages with free access to water and food. One month post-quarantine, the surgical procedure of implanting the prepared neural tissue engineered constructs into the animals was performed.

**Table 1:** Details and implantation locations in experimental animals.

Animal identifier^#^	Gender	Date of birth	Weight at the study start (kg)	Duration of observation	Regions of implantation
40400	Male	Nov. 20 2012	5.4	12 weeks	1. Brain: Parietal lobe 2 cm from the sagittal suture bilaterally. 2. Spinal cord: Lower thoracic T7 – T8 level, posterior surface of the spinal cord in the projection of the posterior spinal column.
40014	Male	Apr. 20 2012	5.6		

#### Surgical protocol

The food supply and water were removed 12 and 4 hours, respectively, prior to the surgical procedure. In order to ensure their safe removal from the cages, animals received an intramuscular injection of Seduxen (1 mg/kg) and Ketamine (10 mg/kg). Their hair was then clean shaved from the back and head, the tibial vein was catheterized, intubation was preformed and the animals were transferred into a sterile operation room, where they were connected to a ventilator with a semi-closed circuit with the following parameters: tidal volume 120–150 mL, respiratory rate 23–26 bpm, FiO_2_ 0.4. Anesthesia was achieved via a bolus dosing of fentanyl and inhalation of isoflurane 1.2–2.0 vol. Prior to the surgery, the animals were also injected with ketonal (10 mg/kg), dexamethasone (1 mg/kg), Tranexam (20 mg/kg) for desensitization and multimodal anesthesia.

#### Implantation into the brain

The skin was scrubbed with iodine and alcohol. An incision of the cutaneous covering was performed followed by a layer-by-layer dissection of the aponeurosis. Using a neurosurgical burr, two milling holes with the diameter of 5 mm were applied in the projection of the parietal fissure, followed by the section of the *dura mater* and dull stratification of the *pia mater* with hemostasis. Using microsurgical forceps, 2–3 neural tissue engineered constructs sized 2×8×0.3 mm were inserted in the brain cortex at a depth of 5–7 mm. Hemostasis was controlled, then the dura mater, aponeurosis and head skin were consecutively sutured. Sterile bandage was applied to the wound.

#### Implantation into the spinal cord

The skin was scrubbed with iodine and alcohol. A 8–10 cm longitudinal midline skin incision was made to expose the T8–11 vertebrae, and paraspinal muscles were separated subperiosteally. A T8 interlaminectomy was performed, and the *dura mater* was exposed, which was dissected longitudinally in the projection of the corresponding segment. 2–3 tissue engineered constructs sized 2×15×0.3 mm were placed on the surface of the spinal cord in the projection of the posterior spinal column and slightly inserted by means of a careful dissection of fibers of the posterior spinal column. The plastic closure of the dura mater was performed with a fascial flap, and the dura mater suture was sealed with fibrin glue. The wound was sutured layer-by-layer and covered with an aseptic bandage.

### Termination and histology

12 weeks after the SPRPix matrix implantation, the animals were anesthetized by an IV injection of ketamine (20 mg/kg), followed by an overdose of propofol administered intravenously. After apnoea and atonic coma took place, the animals’ thorax and mediastinum were dissected, the right atrium auricle was opened for blood removal, the left ventricle was punctured, a cannula was placed transcardially and guided through the aortal valve up to the ascending aorta. Perfusion with 10% buffered formalin solution was carried out though the cannula in a volume equal to the body weight (up to 5 L). Then, the brain and the entire spinal column were separated and fixed for 24 h in the same solution at 4°С. The spinal cord in the projection of the implantation location (plus two segments above and below) was separated from the fixed preparations of the spinal column. The prepared spinal cord and brain samples were dissected along the medial line so that the implanted constructs were both on the right and on the left side. 50 µm-thick sagittal vibratome sections were made from the left preparations and used for immunofluorescent staining and confocal microscopy. Paraffin blocks and 5–6 µm-thick paraffin sections prepared from the other side were used for routine histology studies with cresyl violet (Nissl staining) and hematoxylin-eosin as well as immunohistochemical analysis.

### Immunohistochemistry

Immunohistochemical analysis was performed using thick vibratome sections, as well as paraffin sections, by means of both fluorescent and immunoperoxidase detection. The spinal cord and brain sections were stained using antibodies to Nestin (R&D), SOX2 (BD Biosciences), βIII-tubulin (R&D), MAP2 (Sigma-Aldrich), GFAP (DAKO), NF-200 (Sigma-Aldrich,) macroH2A.1 (ab183041, Abcam), human-specific anti-mitochondria antibody (ab92824, Abcam), and STEM-121 (Y40410, TakaraBio), anti- CD3, CD31 and CD68 (Roche Diagnostics) (all as 5 μg/ml solutions in PBS). In the case of immunofluorescence detection with the use of vibratome sections, Alexa Fluor 488 goat anti-mouse IgG (H + L) and Alexa Fluor 633 goat anti-rabbit IgG (H + L) (all dilutions 1:400; Invitrogen, USA) were used as secondary antibodies, the fluorescence was detected with a Nikon A1 laser scanning confocal microscope (Nikon Co., Japan). Immunoperoxidase staining was performed on thin paraffin sections with a Benchmark ULTRA immunostainer (Ventana, USA) utilizing the manufacturer-recommended detection system.

## Supplementary information

Supplemental materials
