# Supplementary material for: Tissue Engineered Neural Constructs Composed of Neural Precursor Cells, Recombinant Spidroin and PRP for Neural Tissue Regeneration
Source: Sci Rep. 2019 Feb 28;9:3161. doi: 10.1038/s41598-019-39341-9 (PMC6395623; doi:10.1038/s41598-019-39341-9)
Supplement: Supplementary file 1 — Supplemental materials [file 41598_2019_39341_MOESM1_ESM.docx]

**Tissue Engineered Neural Constructs Composed of Neural Precursor Cells, Recombinant Spidroin and PRP for Neural Tissue Regeneration**

**V.P. Baklaushev^1*^, V.G. Bogush^2^, V.A. Kalsin^1^, N.N. Sovetnikov^1^, E.M. Samoilova^1^, V.A. Revkova^1^, K.V. Sidoruk^2^, M.A. Konoplyannikov^1,4^, P.S. Timashev^3,4,5^, S.L. Kotova^4,5^, K.B. Yushkov^6^, A.V. Averyanov^1^, A.V. Troitskiy^1^, J‑E. Ahlfors^7^**

*^1^Federal Research and Clinical Center of Specialized Medical Care and Medical Technologies FMBA of Russia 28 Orekhovy Blvd., 115682 Moscow, Russia*

*^2^* *Scientific Center "Kurchatov Institute" - Research Institute for Genetics and Selection of Industrial Microorganisms”, 1-st Dorozhniy pr., 1, 117545 Moscow, Russia*

*^3^ Federal Research Center "Crystallography and Photonics", Institute of Photonic Technology of the Russian Academy of Sciences, 2 Pionerskaya St., Troitsk, 142190 Moscow, Russia,*

*^4^ Institute for Regenerative Medicine, I. M. Sechenov First Moscow State Medical University, 8 Trubetskaya St., 119991 Moscow, Russia*

*^5^N.N.Semenov Institute of Chemical Physics, 4 Kosygin St., 119991 Moscow, Russia*

*^6^National University of Science and Technology “MISIS”, 4 Leninsky Prospekt, 119049, Moscow, Russia*

*^7^New World Laboratories Inc., Laval, Quebec, Canada*

**Corresponding author:* [*serpoff@gmail.com*](mailto:serpoff@gmail.com)

*
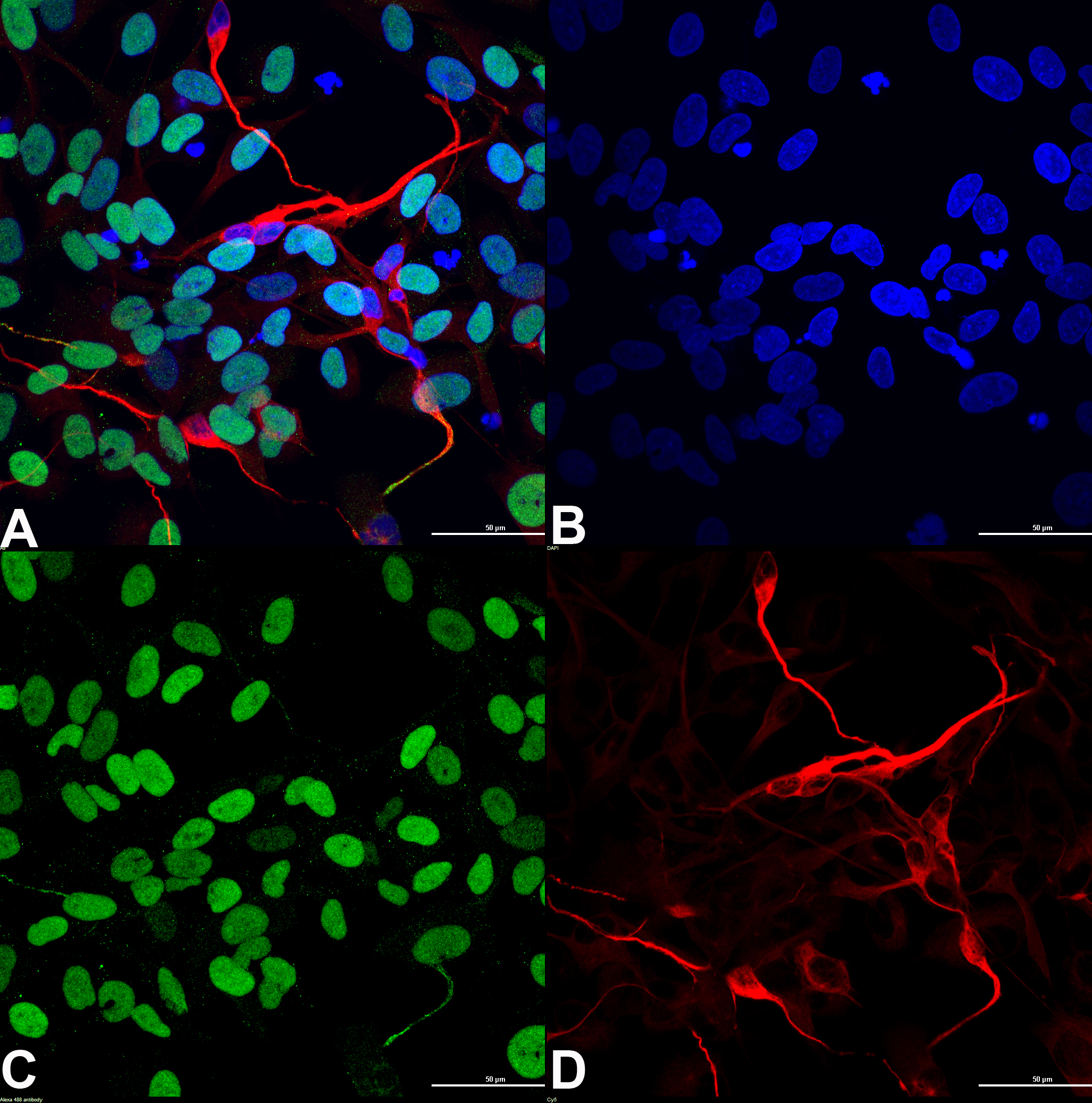
*

Figure 1. Early onset of drNPC-01 culturing on recombinant spidroin rS1/9. A. Merged image. B. Cell nuclei stained by Hoechst C. SOX2 (green fluorescence) and D. βIII-tubulin (red fluorescence). Mouse monoclonal antibodies to SOX2, and goat anti-mouse Alexa Fluor 488 secondary antibodies. Rabbit polyclonal antibodies to βIII-tubulin and goat anti-rabbit Alexa Fluor 633 secondary antibodies. Bar = 50 µm. Laser scanning confocal microscopy.

Figure 2. Neuronal differentiation of drNPC-01 cultured on recombinant spidroin rS1/9. A. BDNF (green) and βIII-tubulin (red). A. Merged image. B. Cell nuclei stained by Hoechst, C. Mouse monoclonal antibodies to BDNF, and goat anti-mouse Alexa Fluor 488 secondary antibodies. D. Rabbit polyclonal antibodies to βIII-tubulin and goat anti-rabbit Alexa Fluor 633 secondary antibodies. E. Enlarged image. Bar = 100 µm.Laser scanning confocal microscopy.

**
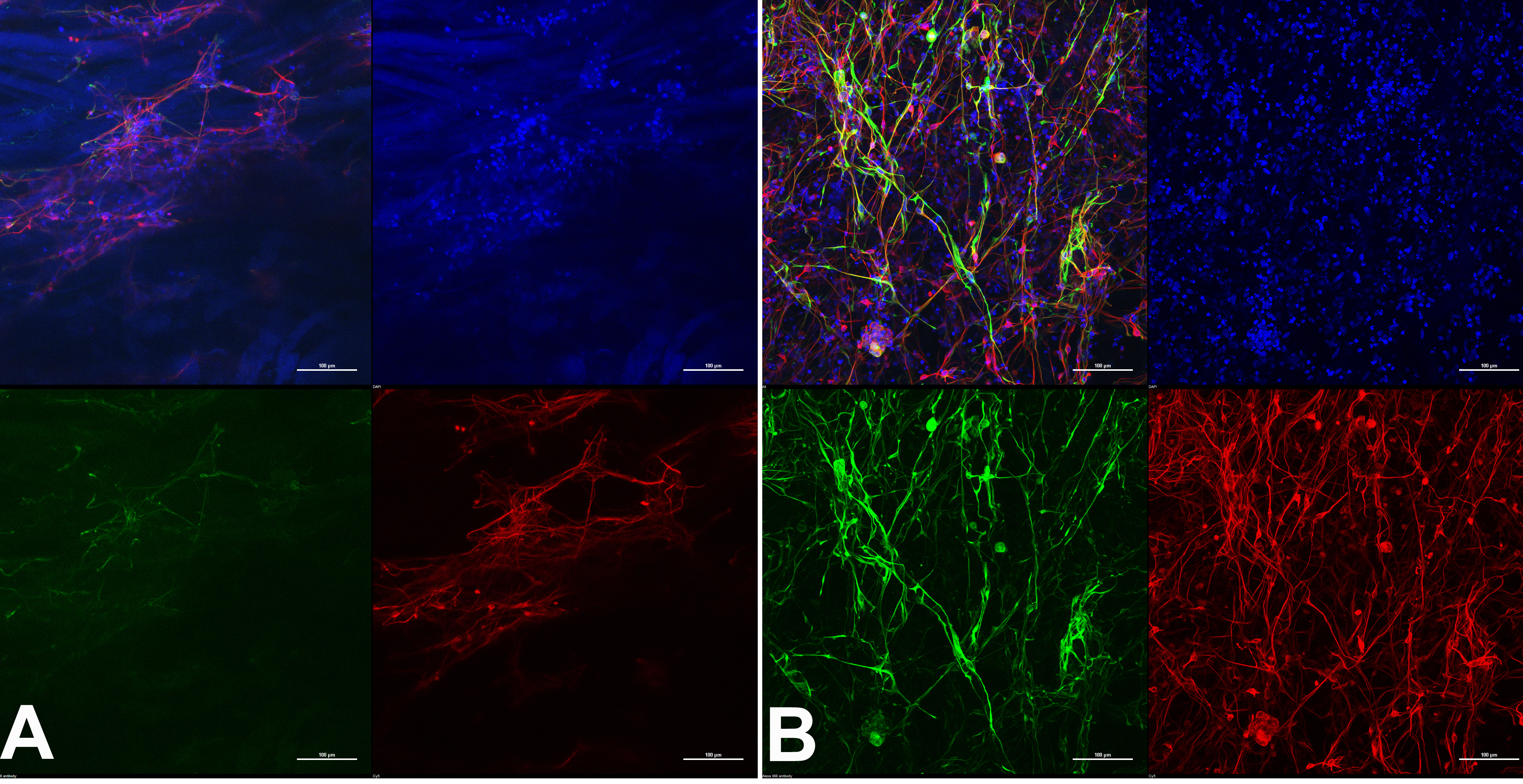
**

Figure 3. drNPC-01 cultured on the Chondro-Gide^®^ collagen scaffold without (A) and with (B) the PRP-based liquid matrix. MAP2 (green); GFAP (red). Cell nuclei were stained with Hoechst. Laser scanning confocal microscopy. Bar =100 µm.


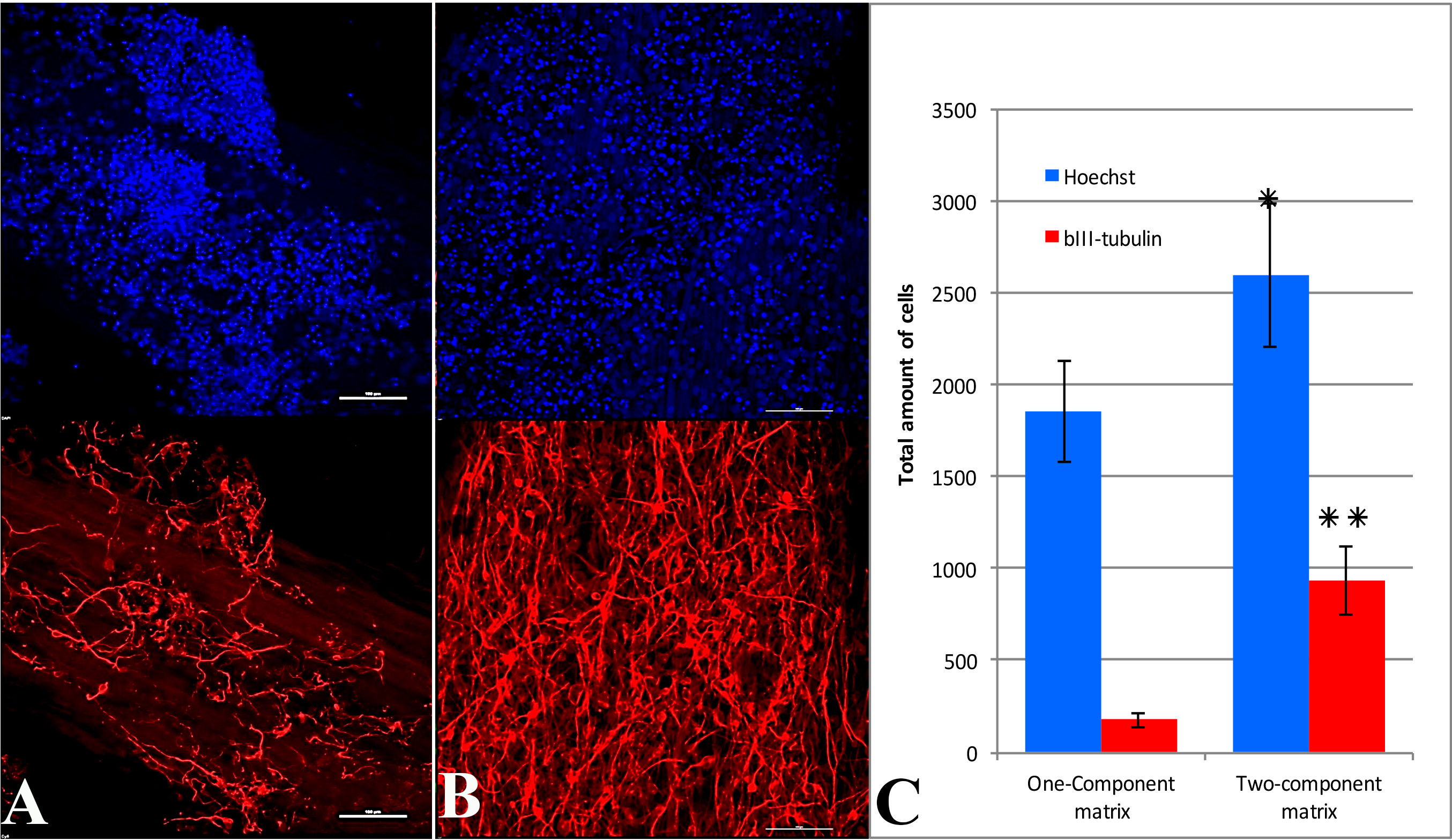


**Figure 4.** SPRPix increases proliferation and neuronal differentiation of drNPCs. NPCs cultured in anisotropic rSS-PCL scaffold only (A) or the two-component SPRPix scaffold (PRP + anisotropic rSS-PCL scaffold) (B).. Hoechst (blue), βIII-tubulin (red). C. Quantification of total cells (blue columns) and βIII-tubulin positive neuronal progenitors (red columns) per 640×640×25µm field (average numbers from 5 repeats).

** - p < 0.01 compared with one-component rSS-PCL scaffold.

* - p < 0.05 compared with one-component rSS-PCL scaffold.

Table 1. Statistical fitting parameters for analyzing the orientation of extended neurites using various FFT-based techniques.

| **Plot ref.** | **Group** | **Imaging method** | **Our fit** | | | | **FiberFit** | | | **ImageJ Directionality plug-in** | | |
| --- | --- | --- | --- | --- | --- | --- | --- | --- | --- | --- | --- | --- |
|  |  |  | ***ɛ*** | ***μ*** | ***σ*** | **R^2^** | ***μ*** | ***σ*** | **R^2^** | ***μ*** | ***σ*** | **Good-ness** |
| A | rSS-PCL scaffold alone | SEM | 0.97 | 80.2° | 27.5° | 0.96 | 79.6° | 12.7° | 0.96 | 77.2° | 9.1° | 0.94 |
| B | drNPCs-01 + SPRPix matrix | ICC MAP2* | 0.79 | 76.0° | 42.6° | 0.93 | 73.7° | 37.6° | 0.81 | 75.0° | 22.3° | 0.92 |
| C | drNPCs-01 + SPRPix matrix | ICC βIII-tubulin | 0.67 | 83.1° | 45.3° | 0.96 | 84.0° | 41.2° | 0.88 | 78.9° | 25.6° | 0.93 |
| D ^(a)^ | drNPCs-01 + rSS-PCL scaffold . | ICC βIII-tubulin | 0.46 | 78.7° | 49.7° | 0.78 | 71.9° | 58.1° | 0.52 | 84.2° | 33.5° | 0.65 |
| E | drNPCs-01 alone (Control  w/o Scaffold) | ICC βIII-tubulin | 0.19 | 38.4° | 52.7° ^(b)^  50.9° ^(c)^ | 0.46 ^(b)^ | 7.3° | 62.4° | 0.12 | 70.3° ^(d)^ | 9.4° ^(d)^ | 0.40 ^(d)^ |

^(a)^ Image pixels with levels below 64 (in 8-bit scale) were set to 0 before further analysis to exclude residual fluorescence of the scaffold present in the original image.

^(b)^ Direct fit with normal distribution did not converge. Values of σ and R^2^ obtained from fitting a normal bivariate distribution with ɛ=0.19.

^(c)^ Varied sample variance directly calculated for distribution of sector intensity sums.

^(d)^ The fit converged to one of the peaks in the multi-peak sample.

* — Immunocytochemical analysis with the previously listed primary antibodies, with the subsequent visualization using secondary antibodies labeled with Alexa Fluor 488/633 by scanning laser confocal microscopy.


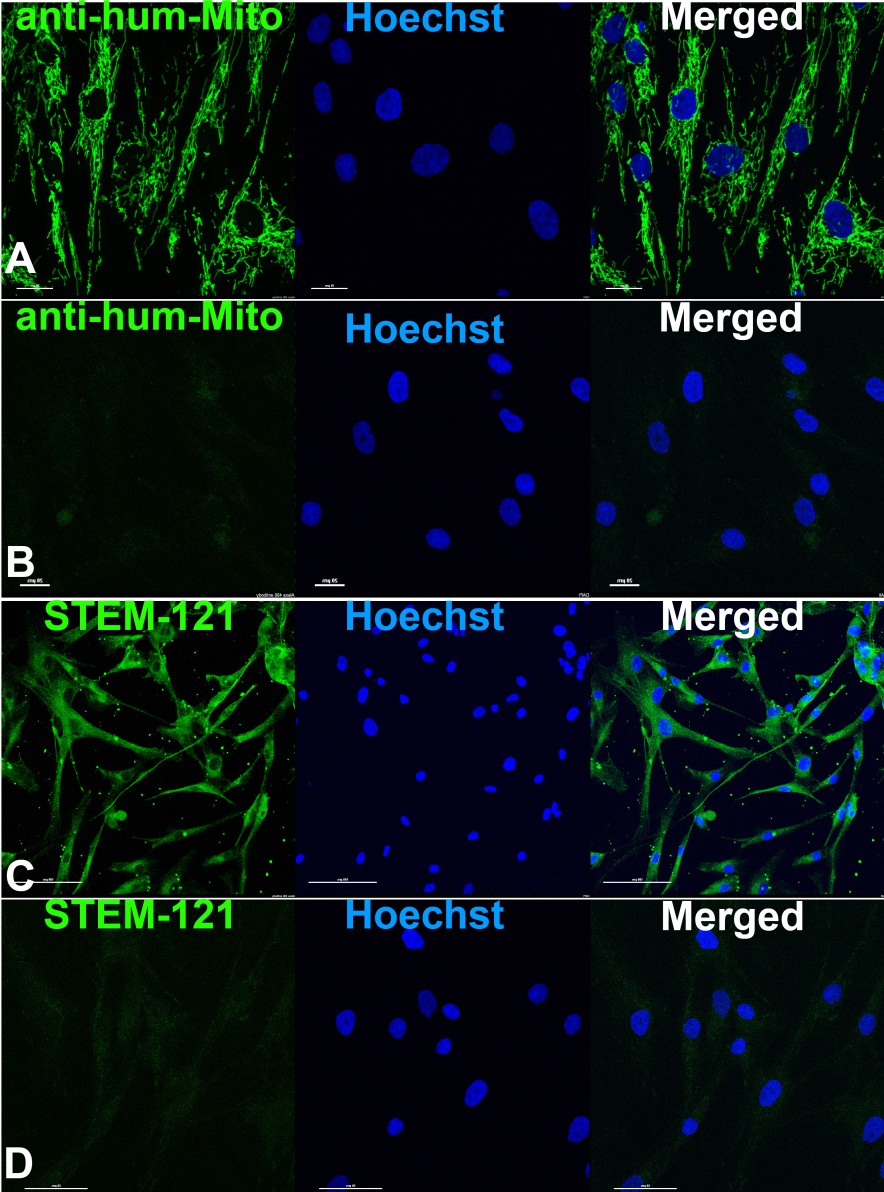


Figure 5. Detection of human cells via human specific antibodies against mitochondria (h-Mito) and cytoplasmic antigen STEM-121. Human (A) and macaca mulatta (B) MSCs stained with h-Mito antibodies. ). Bar size: 20µm. Human (C) and macaca mulatta (D) MSCs stained with STEM-121. Cell nuclei stained by Hoechst (Thermo Fisher Scientific


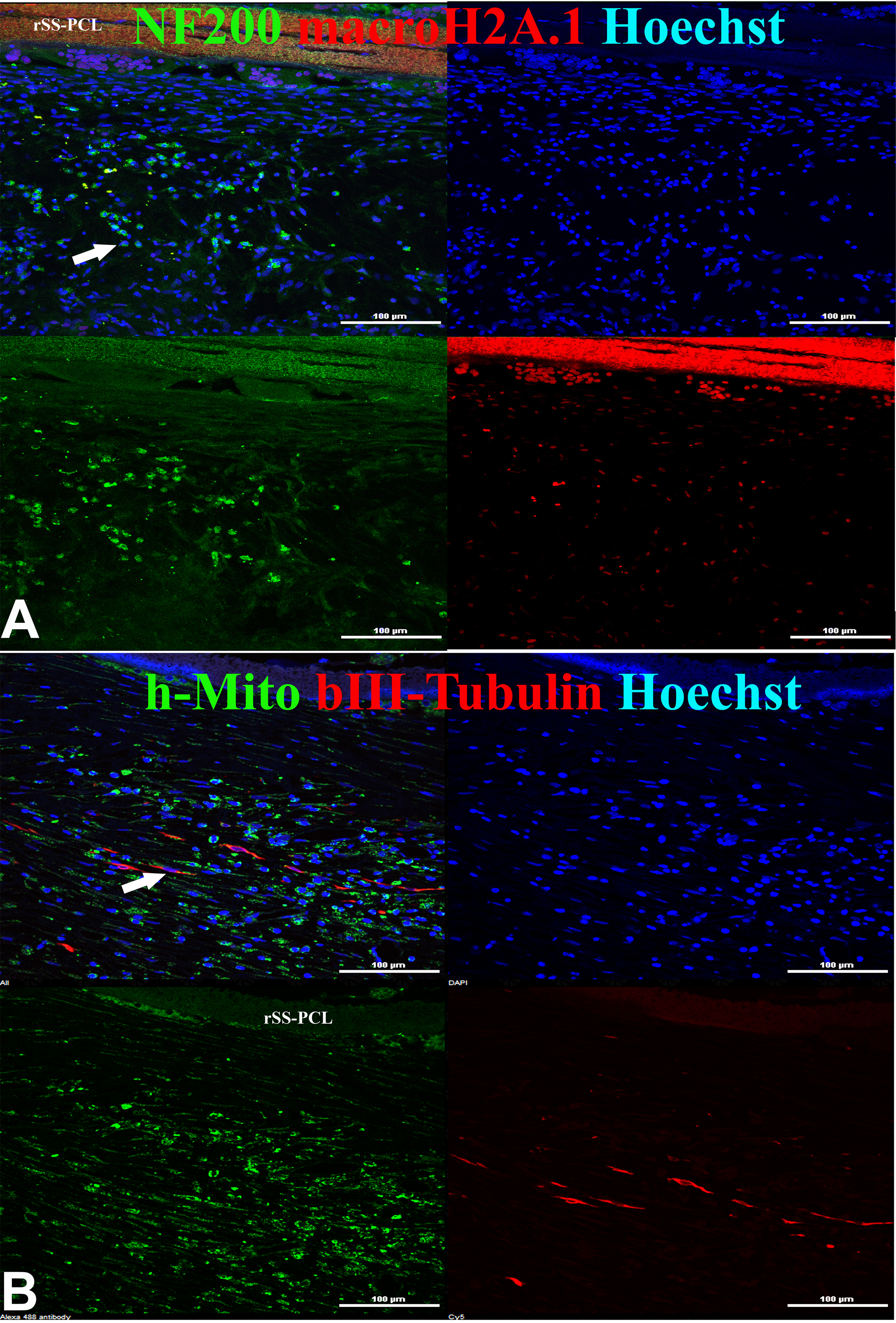


Figure 6. Immunofluorescence analysis of the spinal cord of a rhesus macaque at the site of implantation of the SPRPix matrix with human drNPC-01. ) A. Staining with macroH2A.1 antibodies and anti-NF200 at the site of site of drNPC-SPRPix implantantation (double positive transplanted cells shown by an arrow). B. The same site as in panel A. Co-expression of human specific mitochondrial antigen (h-Mito) and βIII-tubulin (shown by arrow). Bar size = 100µm.
